# KNTC1 introduces segmental heterogeneity to mitochondria

**DOI:** 10.1242/dmm.052063

**Published:** 2025-03-04

**Authors:** Atsushi Tsukamura, Hirotaka Ariyama, Natsuki Hayashi, Satoko Miyatake, Satoko Okado, Sara Sultana, Ichiro Terakado, Takefumi Yamamoto, Shoji Yamanaka, Satoshi Fujii, Haruka Hamanoue, Ryoko Asano, Taichi Mizushima, Naomichi Matsumoto, Yoshihiro Maruo, Masaki Mori

**Affiliations:** ^1^ Department of Pediatrics, Shiga University of Medical Science, Shiga 520-2192, Japan; ^2^ Department of Pediatric Physiology, National Center for Child Health and Development (NCCHD), Tokyo 157-8535, Japan; ^3^ Advanced Pediatric Medicine, Tohoku University School of Medicine, Miyagi 980-0872, Japan; ^4^ Department of Human Genetics, Yokohama City University (YCU) Graduate School of Medicine, Kanagawa 236-0004, Japan; ^5^ Department of Clinical Genetics, YCU Hospital, Kanagawa 236-0004, Japan; ^6^ Research Center for Animal Life Science (RCALS), Shiga University of Medical Science, Shiga 520-2192, Japan; ^7^ Central Research Laboratory, Shiga University of Medical Science, Shiga 520-2192, Japan; ^8^ Department of Pathology, YCU Hospital, Kanagawa 236-0004, Japan; ^9^ Department of Molecular Pathology, YCU Graduate School of Medicine, Kanagawa 236-0004, Japan; ^10^ Department of Obstetrics and Gynecology, YCU Graduate School of Medicine, Kanagawa 236-0004, Japan; ^11^ Department of Rare Disease Genomics, YCU Hospital, Kanagawa 236-0004, Japan

**Keywords:** KNTC1, Glutamine metabolism, Mitochondrial structural heterogeneity, Linear mitochondrial segment, Bent mitochondrial segment

## Abstract

Mitochondria contribute to cellular metabolism by providing a specialised milieu for energising cells by incorporating and processing the metabolites. However, heterogeneity between mitochondria has only partially been elucidated. Mitochondria dynamically alter their morphology and function during the life of an animal, when cells proliferate and grow. We here show that Kntc1, a highly evolutionarily conserved protein, translocates from the Golgi apparatus to linear mitochondrial segments (LMSs) upon glutamine deprivation and plays an essential role in maintaining LMSs. The LMSs to which Kntc1 localised exhibited an increase in the mitochondrial membrane potential, suggesting the role of Kntc1 in functioning as a reservoir for the energy-generating potential. Suppression of Kntc1 led to glutamine consumption and lactate production, thus impacting cellular metabolism, eventually leading to anchorage-independent growth of cells. Indeed, a *KNTC1* variant was identified in a patient with ovarian cancer, suggesting that segmental regulation of the mitochondrial function is essential for maintaining tissue integrity.

## Teaser

**Summary:** Mitochondria possess structural and functional heterogeneities between them, which is established by the glutamine sensor KNTC1

## INTRODUCTION

Mitochondria dynamically change their morphology, which is tightly linked to mitochondrial function (Bulthuis et al., 2019; Pernas and Scorrano, 2016). They provide a specialised, compartmentalised space for cellular metabolism and use various metabolic pathways, such as respiratory energy production, through sequential enzyme reactions that generate adenosine triphosphate (ATP). Through the respiratory chain reactions that convey electrons, a membrane potential forms across the mitochondrial inner membrane, which is than utilised by mitochondrial complex V to synthesise ATP. The regulation of mitochondrial metabolism and electron transfer is traditionally considered to occur throughout the mitochondrion as a unit, and segmental differentiation within mitochondria has yet to be well described. Thus, structural heterogeneity within the mitochondria has yet to be well elucidated.

Mitochondria alter their morphology during different cell cycle phases, under different nutritional conditions (Schrepfer and Scorrano, 2016) and depending on the cell type (Collins et al., 2002). For example, mitochondria take on fragmented and globular shapes during cell division and under nutritionally rich conditions (Picard et al., 2013; Rambold et al., 2011). Cancer cells exhibit fragmented mitochondria due to elevated fission and suppressed fusion activity (Vyas et al., 2016). Mitochondrial fragmentation has also been observed in cells under hyperglycaemic conditions, leading to the overproduction of reactive oxygen species (Yu et al., 2006). By contrast, mitochondria take on an elongated reticular form in differentiated cells that have ceased cell division as well as in cells under nutritionally poor conditions. The reticular shape of mitochondria is associated with protective functions against apoptosis in pancreatic beta cells (Molina et al., 2009) and during mitophagy (Gomes et al., 2011). The mitochondrial reticulum thins when HeLa cells are exposed to galactose (Rossignol et al., 2004). The role of microtubule movement in the maintenance of mitochondrial morphology has also been described (Boldogh and Pon, 2007). Subregional differences in functions and molecular distributions within the mitochondrion are not yet well defined; however, a study assessing mitochondrial membrane potential reported heterogeneity within the mitochondrion regarding membrane potential (Collins et al., 2002).

L-glutamine, a five-carbon amino acid, is one of the most abundant amino acids in cells and the bloodstream (Newsholme et al., 2003). Cancer cells incorporate glutamine faster than normal cells, and their glutamine dependency is utilised as a therapeutic target (Souba, 1993). Cancer cells increase the uptake and utilisation of glutamine depending on their anabolic needs, and exhibit vulnerability to glutamine deprivation, a cellular state known as glutamine addiction (Zong et al., 2016). Cancer cells exhibit unique metabolic properties, known as the Warburg effect (Warburg, 1925); they consume more glutamine as a carbon and nitrogen source than non-tumour cells, while glycolytic metabolism produces lactate (Gao et al., 2009; Moreadith and Lehninger, 1984; Yang et al., 2017). This cellular metabolic state, characterised by higher glutamine consumption, is called glutamine dependency. This glutamine dependency has been observed in cells derived from breast cancer (van Geldermalsen et al., 2016), ovarian cancer (Yang et al., 2014) and pancreatic cancer (Fan et al., 2013). This metabolic uniqueness enables glutamine metabolism as a potential target for cancer therapy (Choi and Park, 2018; Kodama et al., 2020; Leone et al., 2019; Picard et al., 2013).

The usage of glutamine by cancer cells significantly impacts various cellular functions, such as protein synthesis, supply of nitrogen for transamination reactions, incorporation into glutathione and conversion to alpha-ketoglutarate for tricarboxylic acid (TCA) cycle anaplerosis (Cluntun et al., 2017). Acivicin, a glutamine analogue that competitively inhibits glutamine amidotransferase, glutaminase and carbamoyl-phosphate synthetase II (CPSII), has a tumour-suppressive effect and has been tested in clinical trials (Choi and Park, 2018; Weiss et al., 1982; Wise and Thompson, 2010).

Children, unlike adults, harbour exceptional capabilities that allow them to grow, mature, learn, heal, regenerate and adapt (Jam et al., 2018). These child-specific advantages, collectively called juvenility, change depending on age, are more enhanced during the infantile and growing phases, and are gradually lost in adulthood. *Kntc1*, initially identified as a mammalian homolog of *rough deal* (*rod*) in *Drosophila*, is also highly expressed within juvenile organs in mice (Fig. S1A). The *Kntc1* gene is evolutionarily well-conserved among multicellular eukaryotes (Karess and Glover, 1989). The role of KNTC1/ROD has been related to cell division by contributing to the function of kinetochores (Chan et al., 2000; Scaerou et al., 2001). However, the molecular function of Kntc1 needs to be better understood, and the biological relevance of Kntc1 has yet to be described, and almost no reports have been made regarding the molecular functions of Kntc1 in mammalian cells. In this study, starting from its juvenile expression in mouse, we focused on Kntc1 and its role in regulating cellular homeostasis and, unexpectedly, revealed a role of Kntc1 beyond that of kinetochore functioning.

## RESULTS

### Glutamine depletion provokes translocation of Kntc1

**Fig. 1.**
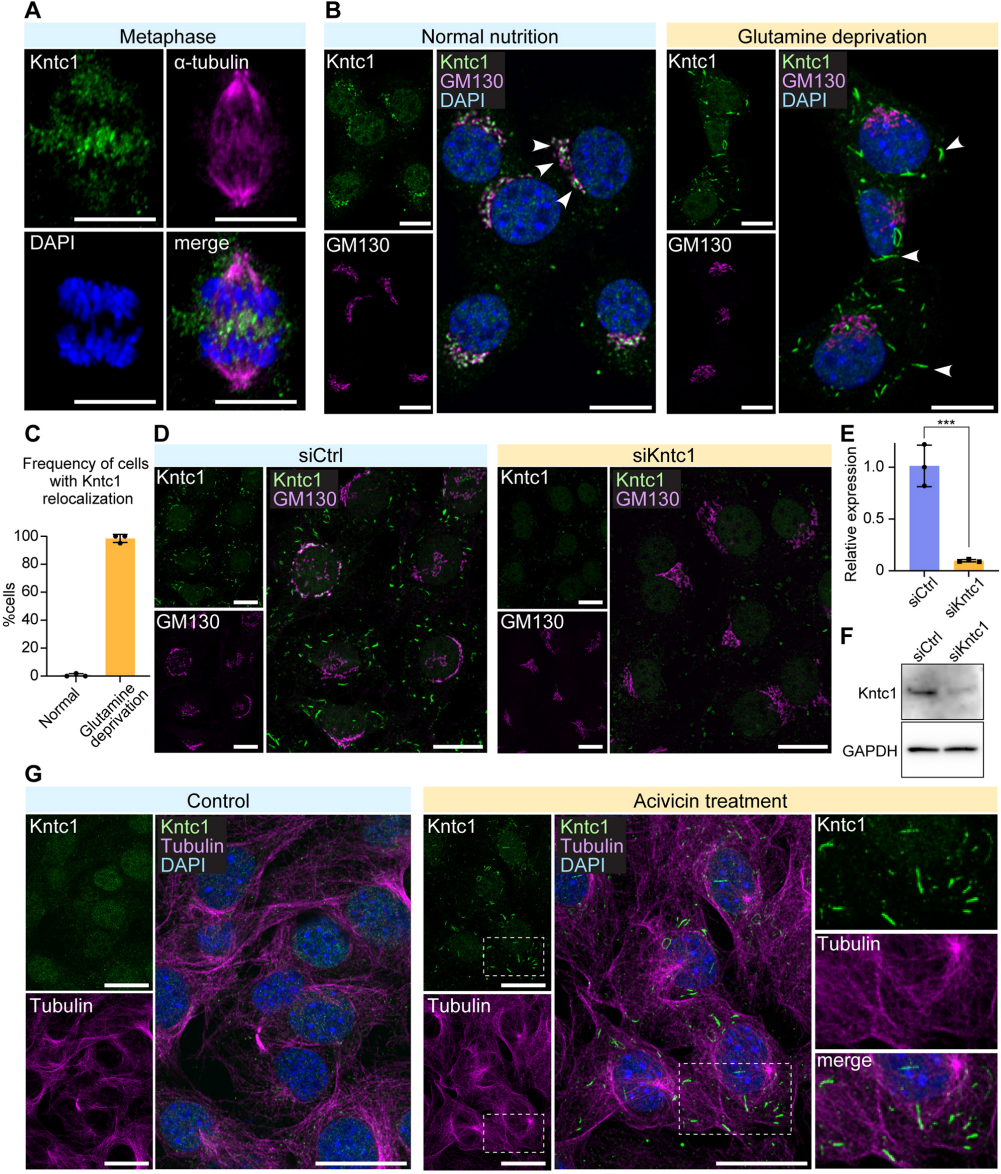
**Kntc1 senses glutamine loss.** (A) Confocal images of dividing NIH3T3 cells immunostained for Kntc1 (green) showing kinetochore-associated localisation of Kntc1. α-tubulin (pink) was stained to visualise the mitotic spindle. Nuclei were stained with DAPI (blue). (B) Kntc1 forms a filamentous structure upon glutamine deprivation for 16 h. Arrowheads indicate Kntc1 signals in GM130-stained Golgi apparatus (normal condition) and the linear structures (glutamine deprivation). (C) Quantification of cells exhibiting the relocalisation of Kntc1 signals. (D) Immunostaining for Kntc1 in NIH3T3 cells transfected with control oligonucleotide or siRNA against Kntc1. Cells were stained with Kntc1 and GM130 antibodies 72 h after the transfection. (E) Real-time qPCR analysis for Kntc1 in NIH3T3 cells transfected with control oligonucleotide or siRNA against Kntc1. RNA was extracted 48 h after transfection. Data were normalised to *Polr2a* and show expression relative to that of siCtrl. (F) Western blot analysis of Kntc1 showing the knockdown efficiency utilising the siRNA against Kntc1. GAPDH was used to show equal loading. (G) The filamentous structure of Kntc1 was induced by treatment with acivicin, which blocks glutamine metabolism. Boxed areas are shown at 1.6× magnification on the right. ***P<0.001, unpaired two-tailed Student's *t*-test. All scale bars: 10 μm

We examined the subcellular localisation of Kntc1 in different cells. As reported previously by Chan et al., (2000), Kntc1 showed accumulation around the metaphase plate in dividing mouse NIH3T3 cells (Fig. 1A). In non-dividing cells, however, Kntc1 localised to the Golgi apparatus, as indicated by its colocalisation with GM130, a cis-Golgi matrix protein, implying that Kntc1 has a more global role in cellular homeostasis (Fig. 1B). We tested several different culture conditions to gain more insight into the biological implication of Kntc1 and found a needle-shaped localisation of Kntc1 in the cytoplasm when cells had been deprived of glutamine (Fig. 1B).

These needle-shaped structures were abolished entirely by siRNA-mediated knockdown of Kntc1 (Fig. 1C), indicating immunostaining specificity that showed excellent knockdown efficiency at mRNA (Fig. 1D) and protein (Fig. 1E) levels. The needle-shaped Kntc1 structures were also provoked by inhibitors of glutamine metabolism such as acivicin (Fig. 1F) and 6-diazo-5-oxo-L-norleucine (DON; Fig. S1B), corroborating that the localisation of Kntc1 is under control of glutamine. Furthermore, the Kntc1 needle-shaped structure was observed in different cell types, such as mouse primary neurons (Fig. S1C), HypoN-E1 hypothalamus-derived cells (Fig. S1D) and Neuro2a neuroblastoma cells (Fig. S1E), indicating that glutamine-dependent localisation of Kntc1 is a general issue.

### Kntc1 localises to the linear mitochondrial segments

**Fig. 2.**
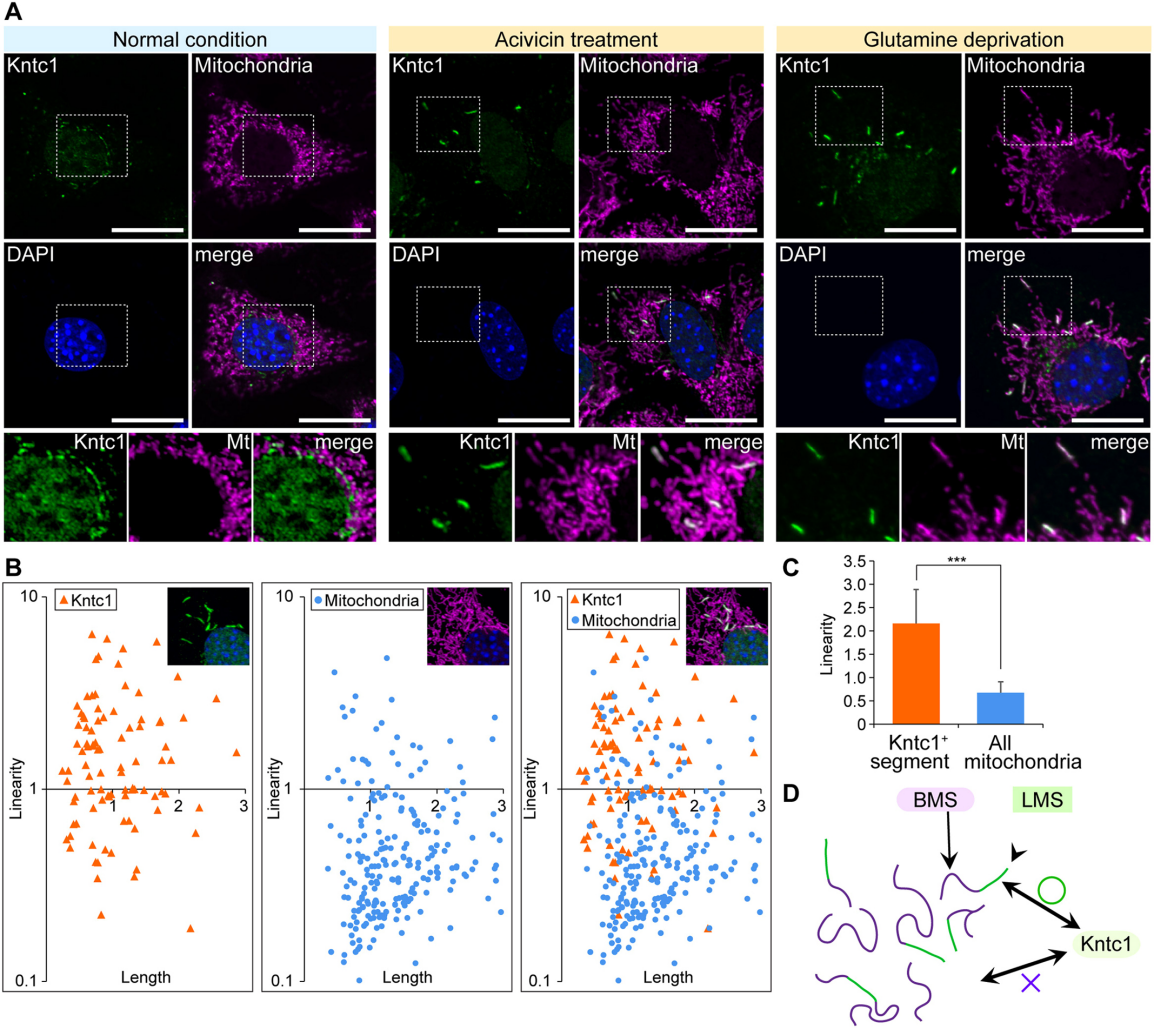
**Kntc1 localises to linear mitochondrial segments.** (A) Kntc1 localises to mitochondria upon glutamine loss in NIH3T3 cells. After treatment with acivicin or glutamine deprivation Kntc1 forms linear segments. Mitochondria were stained with MitoTracker Red CMXRos. Scale bars: 10 μm. Boxed areas are shown at 2× magnification underneath. (B) Kntc1 forms linear segments in mitochondria. The length (in μm) and linearity (reciprocal of curvature) of mitochondria and Kntc1 macrostructures was measured using the Fiji software Kappa. (C) Statistical analysis of linearity (in μm) of the mitochondrial segments that were marked by Kntc1 localisation. (D) Schematic describing preferential localisation of Kntc1 to the linear mitochondrial segment (LMS) but not the bent mitochondrial segment (BMS). ***P<0.001, unpaired two-tailed Student's *t*-test.

Next, we tested whether Kntc1 localised to known subcellular structures and found Kntc1 protein signals to overlap with those of mitochondria (Fig. 2A). We also noticed that Kntc1 proteins did spatially associate with the linear segment of mitochondria – hereafter referred to as linear mitochondrial segment (LMS) – indicating that Kntc1 influences the morphology of mitochondria in a segment-specific manner. Localisation of Kntc1 to the curved subregion − hereafter referred to as bent mitochondrial segment (BMS) − was seldom observed, indicating a relationship between Kntc1 and mitochondrial segmental structures. This structural correlation was assessed by analysis of linearity, in which the mitochondrial segmental structure was measured and calculated as a reciprocal of the curvature. Whereas usual mitochondria showed both bent and linear morphologies, the Kntc1 signals showed high linearity, seldom plotted in areas of low linearity (Fig. 2B). Together, we found that LMSs to which Kntc1 had localised exhibited a highly linear architecture (Fig. 2C), thus providing the unprecedented finding of structural heterogeneity within mitochondria.

### Kntc1 is essential for the formation of linear segments in mitochondria

**Fig. 3.**
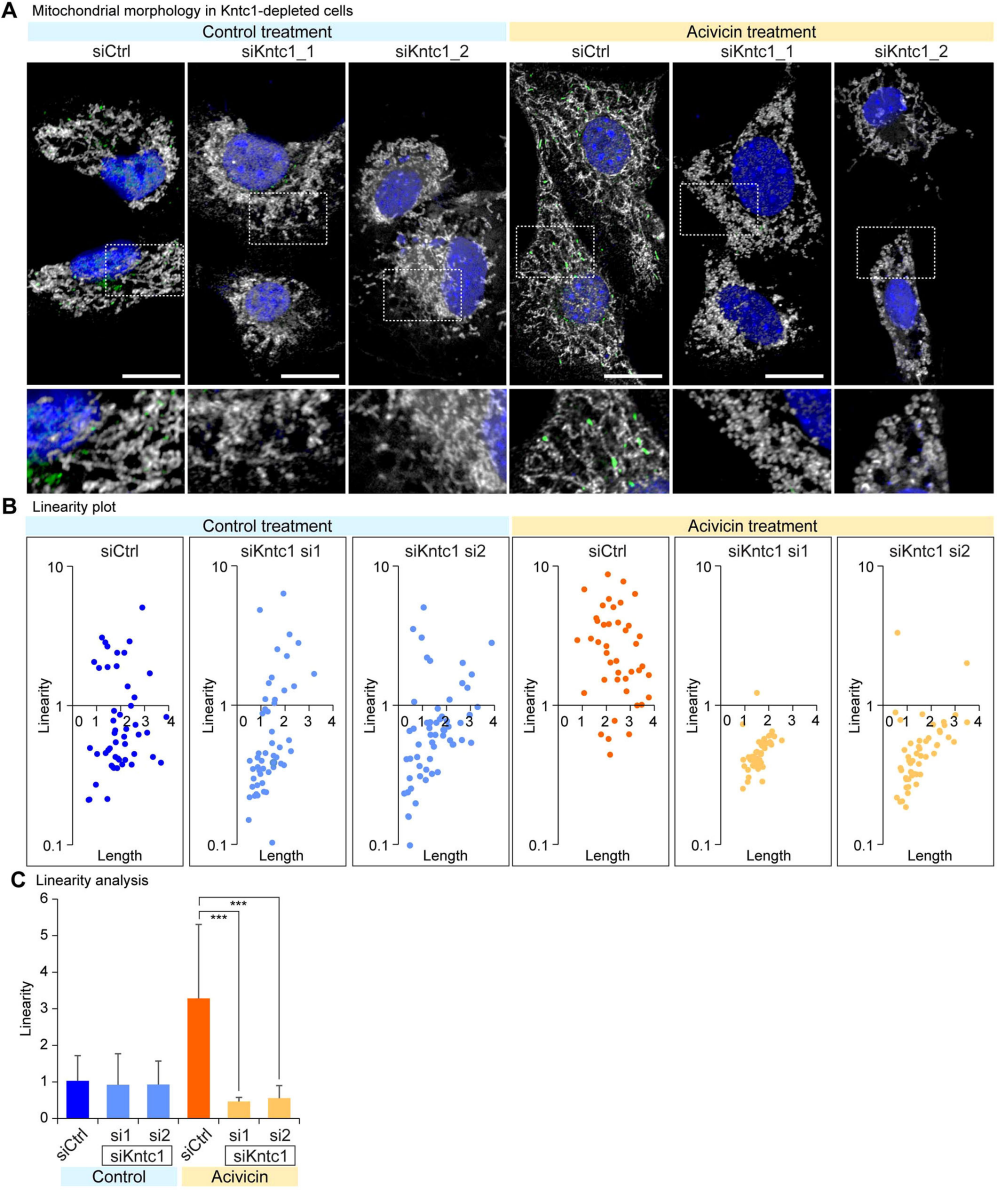
**Kntc1 depletion compromises mitochondrial linearity.** (A) Mitochondrial morphology was assessed in NIH3T3 cells, which were depleted for Kntc1 and treated with acivicin. Mitochondria were stained with MitoTracker Red CMXRos. Nuclei were stained with DAPI. Scale bars: 10 μm. Boxed areas are shown at 2× magnification underneath. (B) Linearity analysis of mitochondria, indicating that Kntc1 is indispensable for the linear mitochondrial structure. (C) Statistical analysis for the segmental linearity in mitochondria. ***P<0.001, one-way ANOVA with post hoc Tukey's test.

To further characterise the role of Kntc1, we interrogated the consequences of Kntc1 depletion on the mitochondrial structures. Acivicin treatment, which recapitulates glutamine deprivation, induced the formation of reticular structures of mitochondria. Depletion of Kntc1 in normal nutritional conditions hardly affected the mitochondrial linearity (Fig. 3A), which is expected considering the localisation of Kntc1 in the Golgi apparatus. However, Kntc1 depletion due to acivicin treatment led to a dramatic change in mitochondrial structure, revealing mitochondrial shortening and failed formation of LMS (Fig. 3A,B). We observed that LMS formation was strongly compromised by Kntc1 depletion, establishing a role of Kntc1 in forming the linear segments in mitochondria. Thus, Kntc1 seems to play a role in the cytoplasm, where it senses glutamine availability and establishes the segmental heterogeneity within mitochondria.

### Kntc1 is necessary for formation of LMSs in a microtubule-independent manner

We further assessed the microstructure of mitochondria by using tomography (Kim et al., 2014). Here, organellar architectures are visualised based on the refractive index that differs within each subcellular compartment. The tomographical observation revealed dynamic changes in mitochondrial shape and reticular networks under acivicin-treated conditions (Fig. S2A). Simultaneous depletion of Kntc1 abrogated the reticular changes of mitochondria (Fig. S2A), corroborating findings obtained with the MitoTracker staining. As also visualised with MitoTracker staining, Kntc1-depleted cells exhibited very round and short mitochondria even after acivicin treatment, confirming that Kntc1 is necessary for the formation of linear segments in mitochondria in response to glutamine deprivation (Fig. S2B).

Various structures, including that of cytoskeleton, regulate the disposition of organelles within cells. Microtubules contribute to the correct positioning of cytoplasmic organelles, such as the endoplasmic reticulum (Terasaki et al., 1986) and mitochondria (Heggeness et al., 1978). Based on the previous report that Kntc1 functions through the kinetochore and microtubule-oriented structures (Chan et al., 2000), we thought of the possibility that Kntc1 influences mitochondrial architecture through microtubules. Acivicin-triggered depletion of Kntc1 did not provoke appreciable changes in microtubules, as examined by staining against α- and β-tubulin (Fig. S2C), and these data demonstrate that regulation of mitochondrial morphology by Kntc1 is microtubule-independent.

### LMS formation involves Mfn1 and Mfn2

**Fig. 4.**
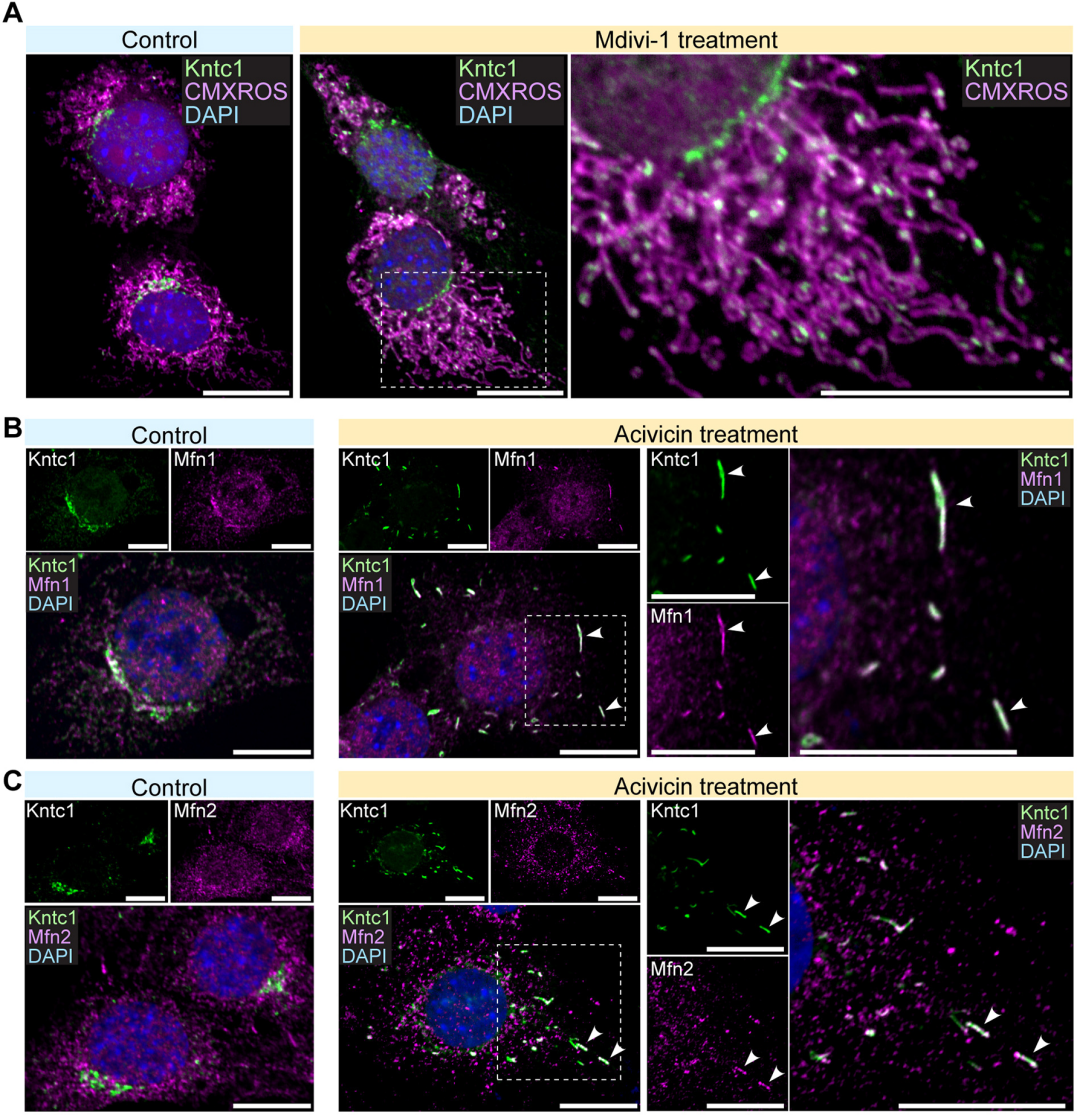
**Kntc1-LMS colocalises with Mfn1 and Mfn2.** (A) NIH3T3 cells were treated with mdivi-1, a DRP1 inhibitor, for 16 h before immunofluorescence analysis against Kntc1. Mitochondria were stained with MitoTracker Red CMXRos. (B) Acivicin treatment of NIH3T3 cells facilitates LMS formation by Kntc1 accompanied by Mfn1 localisation to the LMS. Arrowheads indicate the segments to which Kntc1 and Mfn1 localise. (C) Acivicin treatment of NIH3T3 cells facilitates LMS formation by Kntc1 accompanied by Mfn2 localisation to the LMS. Arrowheads indicate the segments to which Kntc1 and Mfn2 localise. Boxed areas are shown magnified on the right. All scale bars: 10 μm.

To assess the mechanisms underlying the formation of LMS, we tested whether Kntc1 responds to biological cues that induce the macrostructure of mitochondria. By using the mitochondrial division inhibitor mdivi-1 to inhibit the DRP1 mitochondrial fission-inducing protein (Labrousse et al., 1999), the property formation of the mitochondrial network is enhanced (Fig. 4A). Under the mdivi-1-treated conditions, Kntc1 showed localisation to mitochondria, implying involvement of Kntc1 in mitochondrial fusion (Fig. 4A).

To further examine the mechanisms underlying LMS formation, we analysed the localisation of proteins that affect mitochondrial morphologies. Among them, mitofusin 1 (Mfn1; Fig. 4B) and mitofusin 2 (Mfn2; Fig. 4C) showed colocalisation to Kntc1-LMS, revealing the relevance of mitochondrial fusion proteins in the formation of the LMS.

### Localisation of Kntc1 to LMS coincides with enhanced membrane potential

**Fig. 5.**
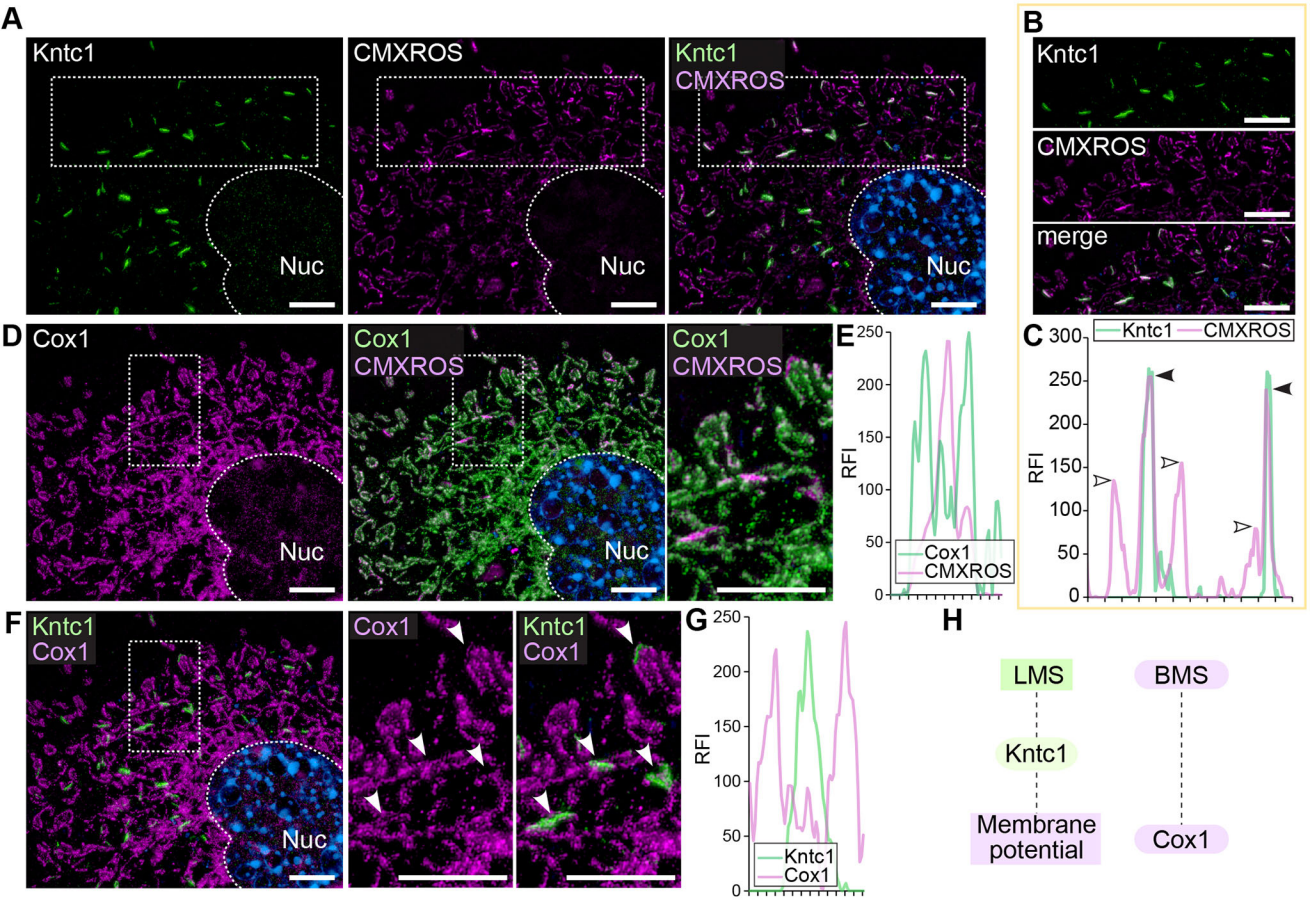
**The LMS harbours a specific metabolic profile.** (A,B) Colocalisation of Kntc1 (green) and mitochondria (magenta) labelled with MitoTracker Red CMXRos (CMXROS) in mouse NIH3T3 cells. Nuc, nucleus. Boxed rectangular areas in A show colocalisation of Kntc1 and MitoTracker Red CMXRox signals and are shown magnified in B. (C) Colocalisation of Kntc1 and MitoTracker Red CMXRos signals reflecting the membrane potential. The intensity of MitoTracker CMXRos is higher in mitochondrial segments to which Kntc1 localises (black arrowheads), which possess higher membrane potential, than in mitochondrial segments to which Kntc1 does not localise (white arrowheads). RFI, relative fluorescence intensity. (D) Colocalisation of Cox1 (green) and CMXRos (magenta). Boxed rectangular area in the middle image shows colocalisation of Cox1 and CMXROS and is shown magnified on the right. (E) Mutually exclusive localisation of Cox1 and mitochondria (CMXRos). (F) Colocalisation analysis of Kntc1 (green) and Cox1 (magenta). Boxed rectangular area is shown magnified in the right image, with arrowheads indicating the Kntc1-localising segment without Cox1 expression. (G) Mutually exclusive localisation of Kntc1 and Cox1. (H) Schematic for the association of LMS with the high membrane potential and not with Cox1. Scale bars: 5 μm. Nuclei were stained with DAPI (blue).

Next, we tested whether Kntc1 proteins located at LMS influence the metabolic function of mitochondria. The Kntc1-associated region of mitochondria showed augmented staining when using MitoTracker Red CMXRos, indicating increased membrane potential (Fig. 5A). The colocalisation analysis showed the LMS to which Kntc1 had localised to exhibit increased intensity of MitoTracker Red CMXRos, indicating that localisation of Kntc1 to LMS (hereafter referred to as Kntc1-LMS) led to enhanced membrane potential (Fig. 5B,C). These findings imply that Kntc1 provoked the segment-specific enhancement of membrane potential. Furthermore, Kntc1-LMS also showed weak levels of Cox1 protein, indicating a distinct molecular composition of Kntc1-LMS (Fig. 5D,E). We observed reciprocal signals of Kntc1 and Cox1 in the acivicin-treated cells, corroborating that Kntc1-LMS harbours a distinct metabolic property (Fig. 5F,G). Thus, Kntc1, upon glutamine deprivation, localises to mitochondria that were bent and induces a linearised structure, which is characterised by its enhanced membrane potential compared to that of the other mitochondrial segments. Also, the reduced expression of Cox1 in Kntc1-LMS suggests its molecularly distinct properties.

### Kntc1 deficiency causes embryonic lethality in mice

The findings obtained through our *in vitro* investigations prompted us to seek relevance in animal models. We, therefore, generated mouse strains harbouring Kntc1 mutation using CRISPR genome editing. An ‘insertion-deletion’ (indel) mutation generated several lines of mice with frameshift mutations, thus, compromising correct expression of Kntc1 protein (Fig. S3A). Further mouse breeding did not result in any further viable homozygous mutants being born, indicating that Kntc1 is indispensable for normal foetal development (Fig. S3B). Further foetal analysis revealed that the homozygous mice were embryonic lethal about the time of implantation, causing absorption embryos. Indeed, the homozygous mutant failed to proceed with organogenesis (Fig. S3B). These findings confirmed that Kntc1 plays an essential role in the early phase of foetal development in mice.

### Kntc1 depletion leads to glutamine overconsumption

**Fig. 6.**
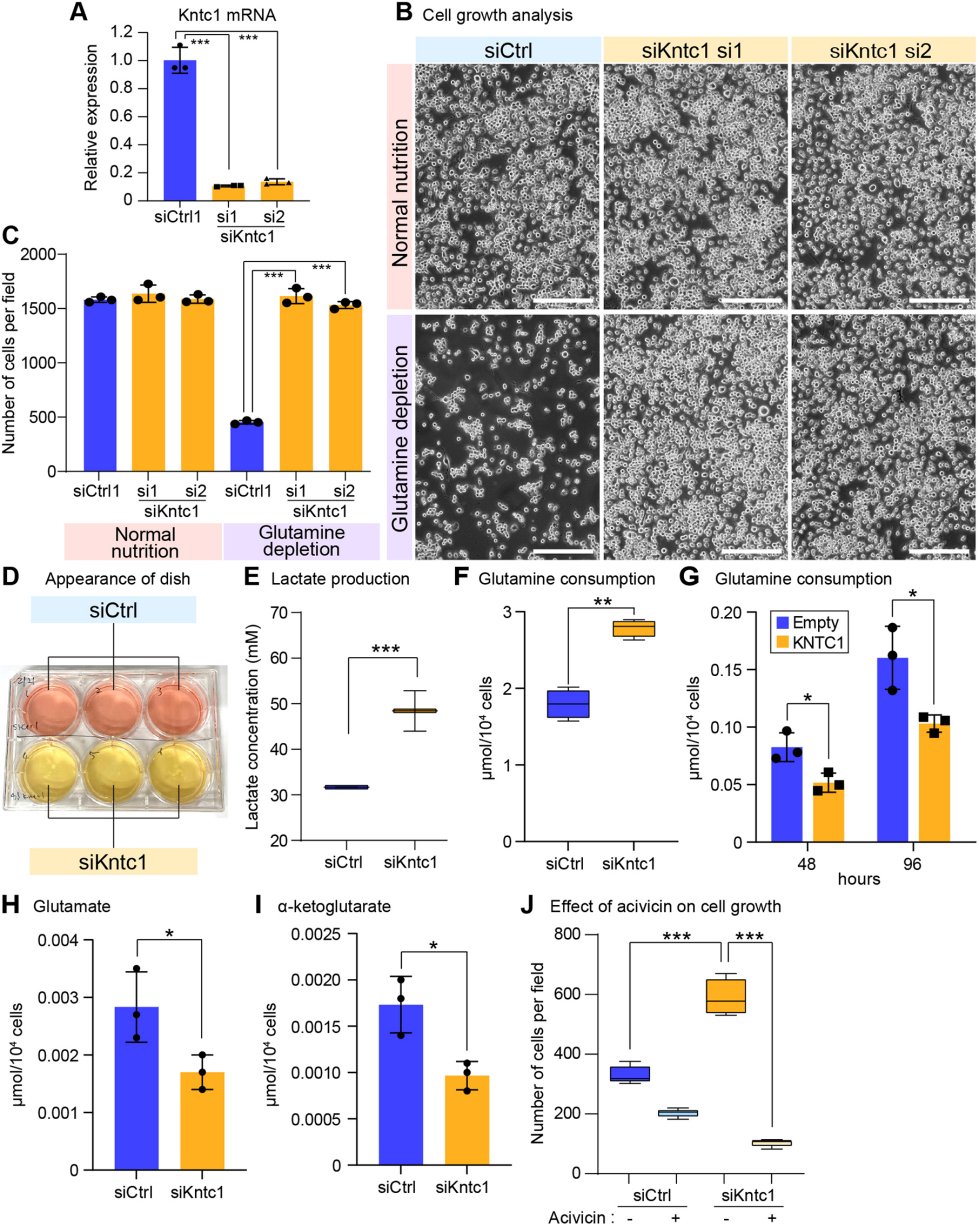
**Kntc1 loss provokes glutamine overuse.** (A) *Kntc1* mRNA levels in Neuro2a cells transfected with siRNA targeting *Kntc1* (siKntc1) or control siRNA (siCtrl). Data were normalised to those of *Gapdh*. (B) Growth analysis of cells that had been depleted of Kntc1 and were grown under normal nutrient conditions (top) or under conditions of glutamine depletion. Scale bars: 200 μm. (C) Quantification of cells described in B. (D) Image of a culture plate of Neuro2a cells transfected with siKntc1 or siCtrl. A change in colour from orange to yellow was observed for the culture medium of Kntc1-depleted cells (bottom). (E) Lactate production analysed in culture medium of siKntc1 or siCtrl cells. (F) Glutamine consumption measured in the culture medium of siKntc1 or siCtrl cells. (G) Glutamine consumption in the culture medium of KNTC1-transfected cells. (H) Glutamate levels measured in siKntc1 or siCtrl cells. (I) α-ketoglutarate levels measured in siKntc1 or siCtrl cells. (J) Assessment of glutamine dependency of Kntc1-depleted cells. The effect of acivicin treatment (+) or control (−) on cell growth was analysed in siKntc1 or siCtrl cells. *P<0.05, **P<0.01, ***P<0.001 unpaired two-tailed Student's *t*-test (A,C,E-I). ***P<0.001, one-way ANOVA with post hoc Tukey's test (J).

The implication that Kntc1 plays a role in regulating mitochondrial architecture prompted us to investigate the role in cell metabolism. We used two different siRNAs to knock down Kntc1 with similar efficiencies (Fig. 6A). The low glutamine condition, which uses 58.4 mg/l of L-glutamine corresponding to 10% of the level under normal conditions, suppressed cell growth of control knockdown cells, whereas Kntc1-depleted cells exhibited higher cell growth even under conditions of glutamine shortage, compared to control knockdown cells (Fig. 6B,C). These observations suggest that Kntc1 suppresses cell proliferation upon glutamine shortage. We noticed that Kntc1 knockdown causes the yellowish change of the culture medium (Fig. 6D), namely acidic changes in the extracellular environment. We measured lactate levels in the culture medium and found that lactate production was facilitated by Kntc1 depletion (Fig. 6E). Furthermore, glutamine incorporation into cells was augmented in Kntc1-depleted cells (Fig. 6F) and suppressed by forced expression of KNTC1 (Fig. 6G), indicating that the role of Kntc1 is to suppress glutamine utilisation. To examine the effect of Kntc1 depletion on glutamine metabolism, we measured the immediate downstream products of glutamine, glutamate and α-ketoglutarate. The analyses revealed a decrease in glutamate (Fig. 6H) and α-ketoglutarate levels (Fig. 6I), supporting that Kntc1 depletion facilitates glutamine consumption.

These findings suggest that Kntc1 suppresses cell proliferation under glutamine-deprived conditions and that Kntc1 depletion provokes glutamine overuse. Based on the finding that Kntc1-losing cells exhibit glutamine-addictive growth status, we examined the effects inhibition of glutamine metabolism has on the overgrowth phenotype. Addition of acivicin to overamplifying cells abrogated the overgrowth phenotype of Kntc1 knockdown cells (Fig. 6J), indicating that the overgrowth of Kntc1-losing cells depended on glutamine metabolism.

### KNTC1 loss is associated with tumorigenesis

**Fig. 7.**
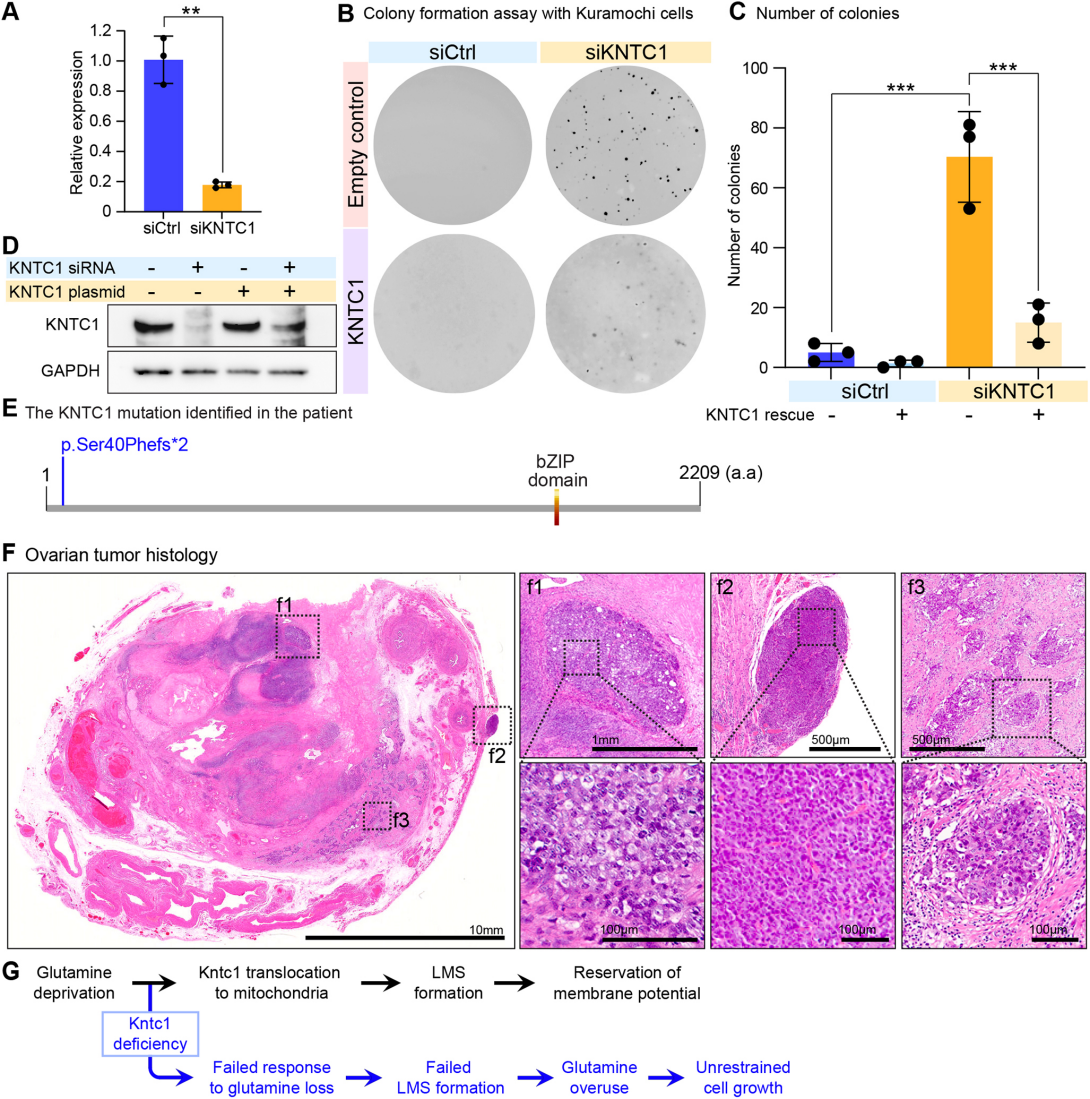
**KNTC1 loss is associated with tumorigenesis.** (A) *KNTC1* knockdown efficiency in Kuramochi cells 48 h after transfection of siRNA targeting *KNTC1* (siKNTC1) or control siRNA (siCtrl). Expression levels of *KNTC1* were normalised to those of *POLR2A*. **P<0.01, unpaired two-tailed Student's *t*-test. (B) Soft agar anchorage-independency analysis performed with siKNTC1 or siCtrl cells as described in A. To rescue the KNTC1 depletion, the KNTC1 plasmid that was mutated so that the *KNTC1* mRNA is not targeted by the siRNA was concomitantly transfected. The soft agar was stained with Crystal Violet to visualise the colonies. (C) Quantification of colony numbers as described in B. ***P<0.001, one-way ANOVA followed by Tukey's multiple comparison tests. (D) Western blot analysis of KNTC1 in Kuramochi cells transfected with siKNTC1 or a *KNTC1*-expressing plasmid. GAPDH is shown as the loading control. (E) Schematic of the genomic structure of *KNTC1* showing the p.Ser40Phefs*2 variant identified in the patient. Usage of the ScanProsite tool identified a basic leucine zipper (bZIP) domain. a.a., amino acids. (F) Histological analysis of ovarian cancer. Boxed areas surround carcinoma cells that formed solid cell clusters within the ovary (f1,f3) or around the fallopian tube (f2), with magnified images on the right. (G) Schematic describing the role of Kntc1 in cell metabolism.

The findings above suggest that Kntc1 depletion caused the cells to exhibit growth potential even if glutamine was limited, a situation in which cell growth is not usually supported. Since overproduction of lactate is a hallmark of tumour cells, we investigated the tumorigenic potential of Kntc1 loss and examined the anchorage-independent growth activity of Kntc1-losing cells. We selected Kuramochi cells, established from human high-grade serous ovarian cancer (Motoyama, 1982), to investigate the effect of KNTC1 depletion on augmenting the tumorigenic property of cells (Fig. 7A). Our assay revealed that KNTC1-depleted cells are capable of growing in an anchorage-independent manner, forming more cell clusters compared to control-depletion cells (Fig. 7B,C). To examine the specificity of siRNA-mediated knockdown, we also tested the effect the rescue of forced expression of siRNA-resistant *KNTC1* gene has in this context (Fig. 7D). Forced expression of *KNTC1* counteracted the overgrowth phenotype provoked by KTNC1 depletion, thereby establishing the specific function of KNTC1 in regulating anchorage-independent growth (Fig. 7C).

A recent nationwide genomic analysis conducted in Japan for undiagnosed diseases (Initiative on Rare and Undiagnosed Diseases; IRUD) identified the frameshift variant, c.119_120del, p.(Ser40Phefs*2), in the *KNTC1* gene of a woman diagnosed with ovarian cancer with histology of adenocarcinoma (hereafter referred to as ‘the patient’). The mutant was predicted to produce a protein comprising 42 amino acids, i.e. being much shorter than the wild-type protein, with 1257 amino acids (Fig. 7E). Pathological analysis of the tumour revealed high-grade serous carcinoma in the bilateral ovary, fallopian tube and peritoneum (Fig. 7F). These clinical findings, combined with cellular transformation assays and cellular metabolism analyses, support the potential role of Kntc1 malfunction in tumorigenesis.

Together, our findings show the unprecedented role of Kntc1 in regulating mitochondrial segmental metabolic functions in a glutamine-sensitive manner. Failed Kntc1 function results in the glutamine-addictive overgrowth phenotype of cells, which was well counteracted by inhibition of glutamine metabolism. Thus, our findings indicate the functional relevance of segmental heterogeneity in the mitochondria, which is essential for maintaining tissue integrity (Fig. 7G).

## DISCUSSION

In this study, we described segmental heterogeneity within mitochondria induced by glutamine shortage in a Kntc1-mediated manner. Our findings indicate that, upon glutamine deprivation, Kntc1 localised from the Golgi apparatus to the LMS, which implies that Kntc1 senses glutamine deprivation and influences mitochondrial functions. A corroborating finding is that inhibition of glutamine metabolism in response to the competitive inhibitors acivicin or DON also provoked the mitochondrial localisation of Kntc1 – an observation that was unexpected because of the known kinetochore-associated relevance of Kntc1. Localisation of Kntc1 to the mitochondria was selectively observed in LMS but not BMS, a finding suggesting that Kntc1 affects the mitochondrial architecture. This idea was supported by our finding that Kntc1 depletion nearly completely abrogated the linear structure of the mitochondria, even in the presence of acivicin.

We found that Kntc1-LMS was localised by Mfn1 and Mfn2, two proteins that induce mitochondrial fusion, suggesting that architectural alteration provoked by Kntc1 involves mitochondrial fusion. This indicates that Kntc1 establishes the LMS by using a fusion-associated mechanism, although it does so by suppression fission. Because Kntc1 does function on the kinetochore, a microtubule-associated chromosomal architecture, we investigated the role microtubules might have in influencing LMS formation. However, we did not find any significant changes in microtubules in Kntc1-depleted cells, which suggest that Kntc1 influences the mitochondrial segmental structure via a different mechanism. A remaining question is how Kntc1 establishes the heterogeneity of mitochondria. We speculate that it is established by accumulating fusion-inducing proteins, i.e. Mfn1 and Mfn2, thus, generating a specialised subregion that harbours distinctiveness in terms of metabolic properties.

Kntc1 depletion was able to induce mitochondrial fragmentation as well as loss of LMS. We assume this is because loss of Kntc1 dysregulates Mfn1 and Mfn2 during formation of LMS, thus, resulting in a fission-predominant state that introduces fragmentation of mitochondria. A similar phenomenon has been reported in previous studies, reporting that depletion of Mitofusin protein causes mitochondrial fragmentation (Jiang et al., 2018; Martorell-Riera et al., 2014).

Kntc1-LMS was concomitantly observed with increased membrane potential. A regional difference in membrane potential has been reported by Collins et al. (2002), in which the intensity of the potentiometric dye tetrachloro-1,1′,3,3′-tetraethylbenzimidazol-carbocyanine iodide (JC-1) was observed to be segmentally different in mitochondria. While we investigated the localisation of Kntc1 to mitochondria, we found that protein levels of Cox1 are lowered in the Kntc1-LMS. This finding implies that the mitochondrial segment to which Kntc1 localises harbours different molecular compositions and metabolic distinctiveness. The important question where exactly within mitochondria Kntc1 is localised to remains, which is crucial in order to elucidate how Kntc1 forms the LMS. Our observations revealed that Kntc1 is a functional determinant of mitochondrial segmentation.

Glutamine is an important energy source, which is converted to glutamate and alpha-ketoglutarate, a substrate of the TCA cycle. Because Kntc1 localisation to LMS was provoked in response to decreasing glutamine levels, Kntc1-LMS might function as a reservoir for the proton gradient utilised to produce ATP. The biological relevance of Kntc1 had initially been suggested by acidification of the culture medium in which Kntc1-depleted cells were grown. Kntc1-depleted cells consumed more glutamine and produced more lactate than control cells (Fig. 6E,F). These findings suggest that the function of Kntc1 is to suppress glutamine consumption when glutamine levels are decreased and to suppress lactate production by avoiding glycolysis. We consider mitochondrial elongation to be a protective measure against a decrease in nutrients, such as glutamine. During glutamine deprivation, Kntc1 relocates to mitochondria, which induces structurally and metabolically distinct subregions and suppresses glutamine consumption to avoid metabolic catastrophe. Without Kntc1, cells fail to form LMS and continue to grow, thereby overconsuming glutamine.

The idea that Kntc1-depleted cells might be glutamine-dependent was assessed by using the glutamine metabolism inhibitor acivicin, which resulted in cell-killing activity. The glutamine-dependent growth of Kntc1-depleted cells suggested that the metabolic transformation occurred due to Kntc1 loss. The agarose anchorage dependency assay revealed that Kntc1-depleted cells harboured more rigorous colony-forming ability than control knockdown cells. Moreover, glutamine metabolism blockade in response to forced expression of KNTC1 abolished the colony-forming activity of Kntc1-depleted cells. These findings indicate that Kntc1 loss elicits the capacity for anchorage-independent growth through a glutamine-dependent metabolic shift.

We also gained insights from clinical findings regarding the patient, and who was diagnosed with ovarian cancer via a pathological diagnosis of high-grade serous carcinoma of the fallopian tube origin. The patient had a heterozygous frameshift variant in *KNTC1*, resulting in a non-functional allele. This implied increased susceptibility to tumorigenesis by the *KNTC1* variation in humans, although involvement of *KNTC1* in tumorigenesis as a germline variant has not yet been reported. As of June 2024, 35 of 941 ovarian cancer specimens (detection rate: 3.72%) had been registered to harbour somatic point mutations within *KNTC1*, with one of 684 showing copy number variations that comprise deletions within *KNTC1* as listed with the Catalogue Of Somatic Mutations In Cancer (COSMIC; https://cancer.sanger.ac.uk/cosmic/gene/analysis?ln=KNTC1). These data suggest that the *KNTC1* germline variant has caused the occurrence of the ovarian tumour in the patient, although future studies are required to determine the total contribution of KNTC1 variation to cancer. A study by Liu et al. has shown that KNTC1 protein levels are enhanced in non-small-cell lung cancer tissues compared to levels in adjacent normal tissues (Liu et al., 2022). This finding implies that not only reduced but also increased levels of KNTC1 are detrimental to maintaining the normal cell state. A possibility is that properties of KNTC1 in tumorigenesis are tissue-dependent, since the hippo-YAP pathway − which is densely connected to tumorigenic machinery − shows a distinct tendency in the context of intestinal tumorigenesis (Barry et al., 2013).

A recent study by Ryu et al. has reported that subpopulations of mitochondria are induced by energy demand (Ryu et al., 2024). Further investigations into mitochondrial heterogeneity will pave the way to better understanding how the cells within an organism cope with changes in environment.

Overall, our observations suggest that Kntc1 senses glutamine shortage and influences mitochondrial functions by shifting the segmental properties of mitochondria. Kntc1-LMS harbours unprecedented properties in terms of membrane potential and molecular expression, and Kntc1 malfunction leads to a gain in anchorage-independent growth capacity through a metabolic shift towards glutamine dependence.

## MATERIALS AND METHODS

### Establishment of *Kntc1* mutant mice

The institutional animal care approved all mouse experiments and use committees at the Shiga University of Medical Science and the National Centre for Childhood Health and Development (NCCHD). All animal experiments were performed following the relevant guidelines and regulations.

### Reagents for genome editing

The *Kntc1* mutant mouse strains were generated using CRISPR-Cas9 technology as described previously (Hibino et al., 2022; Kadota et al., 2020). Guide RNA (gRNA) was designed using CRISPRdirect (https://crispr.dbcls.jp/). The DNA sequence 5′-ACGAGAGTCAGCGAACGTACCGG-3′ was cloned into the gRNA cloning vector (Addgene Plasmid ID #41824) to express gRNA against mouse Kntc1. For gRNA synthesis, the DNA sequence of the T7 RNA polymerase recognition site was added to the gRNA sequence by the polymerase chain reaction (PCR). PCR products were purified and used as the template for *in vitro* RNA synthesis by using the mMESSAGE mMACHINE T7 Transcription Kit (cat. no.: AM1344, ThermoFisher Scientific). The synthesised gRNA was purified by using MEGAclear (cat. no.: AM1908, ThermoFisher Scientific). Recombinant Cas9 protein was purchased (GeneArt Platinum Cas9 Nuclease, cat. no.: B25642, ThermoFisher Scientific).

### Microinjection

Mouse zygotes were obtained by *in vitro* fertilisation (IVF) of WT C57BL6/N gametes, and gRNA (100 ng/ml) and Cas9 protein (30 ng/ml, TrueCut Cas9 Protein v2, cat. no.: A36497, ThermoFisher Scientific) were mixed and microinjected into the pro-nuclei and cytoplasm of zygotes. Injected embryos were then incubated at 37°C until they were transferred into pseudo-pregnant females at the two-cell stage.

### Genotyping and breeding

Genomic DNA was extracted from the tail tips of pups, and the genomic sequence around the gRNA target site was PCR-amplified using forward primer 5′-TGGGTTGCTATCAGCTCTCC-3′ and reverse primer 5′-TGAATTTCAGGCCAGTCTCA-3′. The PCR products were treated with ExoSAP-IT Express (75001.40.UL, ThermoFisher Scientific) and sequenced with the forward primer.

### Cell culture

Neuro2a cells were cultured in Eagle's minimal essential medium (MEN) supplemented with 10% foetal bovine serum (FBS). NIH3T3 and HypoN-E1 cells were cultured in Dulbecco's modified Eagle's medium (DMEM; 08458-16, Nacalai Tesque, Japan) containing 10% FBS and penicillin/streptomycin (08458-16, Nacalai Tesque). Cells of the human Kuramochi cell line were obtained from JCRB Cell Bank (National Institutes of Biomedical Innovation, Health and Nutrition, Japan) and cultured in RPMI1640 medium (30264-85, Nacalai Tesque) containing 10% FBS and antibiotics (penicillin/streptomycin). Following standard sterile procedures, neuronal cells were isolated from mouse fetal brain at embryonic stage (E)16.5 to E18.5. Embryonic mouse brains were dissected in cold HBSS (cat. no.: 085-09355, Wako, Japan). Cortical tissues were minced and digested for 5 min with 0.25% Trypsin-EDTA and DNase I at 37°C. The digestion was stopped with DMEM containing 10% FBS. Cells were centrifuged (500 ***g***, 10 min) and resuspended in Neurobasal medium (A35829-01, ThermoFisher Scientific, MA) with B27 (cat. no.: 17504044, ThermoFisher Scientific), Glutamax (cat. no.: 35050061, ThermoFisher Scientific), and penicillin/streptomycin (penicillin, 100 units/ml; streptomycin, 100 µg/ml), then triturated to a single-cell suspension. Cells were plated on poly-D-lysine-coated plates and incubated at 37°C under 5% CO_2_. After 4-6 h, the medium was replaced, and cultures were re-fed every 2-3 days until the immunostaining analysis was done.

For glutamine deprivation, low (58.4 mg/l) glutamine condition was achieved by culturing cells for 16 h in low-glutamine culture medium, that is a 1:9 mix of DMEM containing L-glutamine at 584.0 mg/l with glutamine-free DMEM (cat. no.: 11584-85, Nacalai Tesque, Japan) at 1:9. To block glutamine metabolism, cells were incubated in the presence of acivicin (cat. no.: 14003, Cayman Chemical, MI, USA) at 2 mM or 6-diazo-5-oxo-L-norleucine (DON; cat. no.: 045-32441, Fujifilm Wako, Japan) at 2 mM for 24 h. The fusion-dominant status of mitochondria was induced by culturing cells in the presence of mdivi-1 (cat. no.: S7162, Selleck Chemicals, Houston, TX, USA) at 20 μM for 16 h.

For knockdown experiments, Lipofectamine RNAiMAX (cat. no.: 13778150, ThermoFisher Scientific) was used to transfect the siRNA duplexes at 10 nM: mouse *Kntc1* siRNA (Sigma-Aldrich, siRNA ID SASI_Mm01_00173016, SASI_Mm01_00173017, SASI_Mm01_00173018), human *KNTC1* siRNA (SASI_Hs01_00027327) and negative control oligo (AM4611, ThermoFisher Scientific), with all three different siRNAs showing similar results. Overexpression KNTC1was done by using the KNTC1-expressing plasmid pFN21A-KNTC1 (Product ID: FHC05777) obtained from the Kazusa DNA Research Institute. To construct the siRNA-resistant KNTC1 expression plasmid, synonymous mutations were introduced to the sequence containing the siRNA-target site, 5′-CAGTTAGAACTTCCGGCT T-3′, to generate the sequence 5′-CAGTTAGAGCTGCCAGCCT-3′. The knockdown efficiency was assessed by real-time qPCR and western blot analyses at the indicated time point.

Sequences of primers used are as follows: human *KNTC1*, 5′-ATGCATGCGATGAATACCAA-3′ and 5′-TTCACAGGGAGCAGTGTCTG-3′; human *POLR2A*, 5′-GGGTGGCATCAAATACCCAGA-3′ and 5′-AGACACAGCGCAAAACTTTCA-3′; mouse *Kntc1*, 5′-CCTGTGAGATCCTGCAGACA-3′ and 5′-AATTAGCGGCAGACTCCAGA-3′; mouse *Polr2a*, 5′-GAGTCCAGAACGAGTGCATGA-3′ and 5′-ACAGGCAACACTGTGACAATC-3′.

The cellular growth was assessed by counting the number of cells per field 72 h after transfection. Phase-contrast images of the cells were taken with an EVOS FL microscope (ThermoFisher Scientific).

### Mitochondrial morphological analysis

Linearity, length and average linearity (linearity divided by length) were measured by using the Fiji plugin for Curvature Analysis (Mary and Brouhard, 2019 preprint). Linearity was defined as the reciprocal of the curvature, which is calculated using Kappa. For each condition, four to eight cells were quantified. The images obtained by confocal microscopy were used.

### Tomography

Tomography was performed with Tomocube HT-X1 (Tomocube, Korea) according to the manufacturer's instructions. Briefly, cells transfected with siRNA control or against Kntc1 were treated with H_2_O or acivicin for 24 h, and refractive index-based visualisation of organelles was performed at least in triplicate. To corroborate the identification of mitochondria, we used MitoTracker Red CMXRos (cat. no.: M7512, ThermoFisher Scientific) and observed its signals in the same dishes.

### Cell metabolism

According to the manufacturer's instructions, we quantified glutamine with Glutamine Assay Kit-WST (cat. no.: G268, Dojindo, Japan), glutamate with Glutamate Assay Kit-WST (cat. no.: G269, Donjindo), α-ketoglutarate with α-Ketoglutarate Quantitation Kit (cat. no.: MAK541, Sigma-Aldrich) and lactate with Lactate Assay Kit-WST (cat. no.: L256, Dojindo). For glutamine and lactate, conditioned medium used to grow Neuro2a cells depleted or not for Kntc1 was sequentially recovered during the time course. After dilution of the supernatants, so that the concentration of the target metabolites was within the standard curve, concentration of glutamine and lactate was measured using each kit. Experiments were repeated three times, and results shown as the mean±standard error of the mean (±s.e.m.). For glutamate and α-ketoglutarate, cell lysates were subjected to the measurement according to the manufacturer's instructions.

### Anchorage dependency assay

Cells of the Kuramochi cancer cell line were suspended in RPMI1640 with 10% FBS and 0.3% Agarose XP (cat. no.: 312-06512, Nippon Gene, Japan) and plated at 10,000 cells/well in six-well culture dishes on a layer of 0.6% agar containing the same medium. RPMI1640 with 10% FBS was added to the gels. Cell colonies were stained with 0.005% Crystal Violet in PBS after 10 days in culture and quantified using NIH ImageJ.

### SDS-PAGE and western blot analysis

Kuramochi cells were harvested in RIPA lysis buffer containing 25 mM Tris-HCl pH 7.6, 150 mM NaCl, 1% NP-40, 1% sodium deoxycholate and 0.1% SDS. Lysates were centrifuged at 20,400 ***g*** for at least 5 min to remove debris. Then, 5× Laemmli sample buffer was added to the lysates, followed by boiling at 95°C for 2 min. Protein samples (10 μg per lane) were separated using SDS-PAGE and transferred to a Hybond-P PVDF membrane (GE Healthcare). Western blotting was performed using the following antibodies: anti-Kntc1 polyclonal (Invitrogen, cat. no.: PA5-40762, 1:1000) and rabbit monoclonal anti-GAPDH (Cell Signaling Technology, cat. no.: 5174, dilution 1:2000). Secondary antibody used was goat anti-rabbit IgG conjugated to horseradish peroxidase (Thermo Fisher Scientific, cat. no.: 32460; dilution 1:1000). Immunoreactive bands were detected by using Chemi-Lumi One Super (cat. no.: 02230-14) or Chemi-Lumi One Ultra (cat. no.: 11644-24) both Nacalai Tesque, Japan.

### Immunofluorescence staining

For immunostaining of cultured cells, cells were fixed with 4% paraformaldehyde (PFA) at room temperature (RT) for 10 min and permeabilised with 0.1% Triton X-100 in phosphate-buffered saline (PBS) for 2 min. For blocking, cells were incubated with 2% foetal bovine serum (FBS) in PBS for 1 h at RT. Cells were then incubated at 4°C overnight with primary antibodies against Kntc1 (cat. no.: PA5-40762, Invitrogen, 1:200), Mfn1 (cat. no.: D-10, sc-166644, Santa Cruz Biotechnology), Mfn2 (cat. no.: F-5, sc-515647, Santa Cruz Biotechnology), Cox1/mt-Co1 (cat. no.: ab14705, Abcam, 1:400), GM130 (cat. no.: 610823, BD Biosciences, 1:400), α-tubulin (cat. no.: 3873, Cell Signaling Technology, 1:400), and β-tubulin (cat. no.: 86298, Cell Signaling Technology, 1:400). After washing with PBS, cells were incubated with anti-mouse IgG conjugated to Alexa Fluor 546 (cat. no.: A11030, Invitrogen, 1:1000) or anti-rabbit IgG conjugated to Alexa Fluor 488 (cat. no.: A11008, Invitrogen, 1:1000) for 1 h at RT. Mitochondria were stained with MitoTracker Red CMXRos (cat. no.: M7512, ThermoFisher Scientific). Prolong Diamond Antifade mountant with DAPI (cat. no.: P36971, ThermoFisher Scientific) was used for mounting and nuclear staining. Cellular images were taken with a confocal microscope (FLUOVIEW FV3000, Evident, Japan).

### Human analysis

#### Study participant

The Committee approved the study for Ethical Issues at Yokohama City University School of Medicine. The participant or the family were informed about the purpose of this research and signed a written informed consent.

#### Genomic analysis

As previously described, whole-exome sequencing was performed using genomic DNA extracted from leukocytes obtained from the patient at Yokohama City University (Sekiguchi et al., 2019). Briefly, exonic DNA was captured by using the SureSelect Human All Exon V6 kit (Agilent Technologies, Santa Clara, CA, USA) and sequenced on an Illumina HiSeq2500 (Illumina, San Diego, CA, USA) with 101-bp paired-end reads. Image analysis and base calling were performed using sequence control software with real-time analysis and CASAVA software (Illumina). Reads were aligned to GRCh37 by using NovoAlign. The marking of PCR duplicates, indel realignment, and base quality score recalibrations were performed using Picard and the Genome Analysis Toolkit (GATK). Variants were called by the GATK Unified Genotyper (http://www.broadinstitute.org/gatk/) and annotated using ANNOVAR after excluding common variants registered in the common dbSNP135 database (minor allele frequency≥0.01). Detected variants were confirmed by Sanger sequencing. The functional domain in human KNTC1 protein was searched using the ScanProsite tool (de Castro et al., 2006).

### Statistical analysis

All the biological experiments were repeated at least three times to assess reproducibility. The presented data were collected from biologically independent samples. Statistical analysis was performed using GraphPad Prism v.9.0 (GraphPad, MA). All data are represented as mean±s.e.m. unless otherwise specified. Unpaired Student's *t*-tests were used for comparisons between two groups. To compare several groups, data were initially analysed using one-way analysis of variance (ANOVA) to determine any significant differences between the groups. Post-hoc comparisons were conducted using Tukey's multiple comparisons test to identify specific group differences. The statistical parameters and mouse numbers used per experiment are specified in the figure legends. Statistical methods were not used to predetermine the sample size. *P*<0.05 was considered to be significant throughout the study.

### Clinical summary

All clinical investigation was conducted according to the principles expressed in the Declaration of Helsinki. The patient was a 62-year-old female at the time of diagnosis. Her mother had been diagnosed with malignant peritoneal mesothelioma at the age of 75 and died 6 months later. Since her mother had died during our study, genomic analysis regarding *KNTC1* could not be performed. The patient had Bland-White-Garland syndrome with an anomalous left coronary artery originating from the pulmonary artery, which had been surgically treated in her 50s. She visited the hospital with complaints of lower abdominal pain. A pelvic tumour, peritoneal dissemination and lymph node metastasis were found, and she was diagnosed with ovarian cancer FIGO stage IIIC. After three cycles of chemotherapy with paclitaxel and carboplatin, she underwent a simple hysterectomy, bilateral adnexectomy and oophorectomy but the disease recurred just after six cycles of post-operative chemotherapy. Despite second-line chemotherapy with doxorubicin hydrochloride, she passed away one year after the onset of ovarian cancer. Pathological analysis revealed neoplastic lesions in the bilateral ovaries, fallopian tubes, uterine serosa, omentum and sigmoid colon. The tumour exhibited high-grade atypia with solid nests and focal necrosis, likely due to chemotherapy. Immunohistochemistry showed nuclear expression of estrogen and progesterone receptors, and transcription factors PAX8 and WT1 in tumour cells, but was negative for the cellular tumour antigen p53 (null type), consistent with high-grade serous carcinoma. Since the fallopian tube had been involved, the tumour was − according to the WHO classification of Tumours (2020) − considered to have originated from the fallopian tube.

## Supplementary Material

10.1242/dmm.052063_sup1 Supplementary information
