# Supplementary material for: KNTC1 introduces segmental heterogeneity to mitochondria
Source: Dis Model Mech. 2025 Mar 4;18(3):DMM052063. doi: 10.1242/dmm.052063 (PMC11911638; doi:10.1242/dmm.052063)
Supplement: Supplementary information [file dmm-18-052063-s1.pdf]

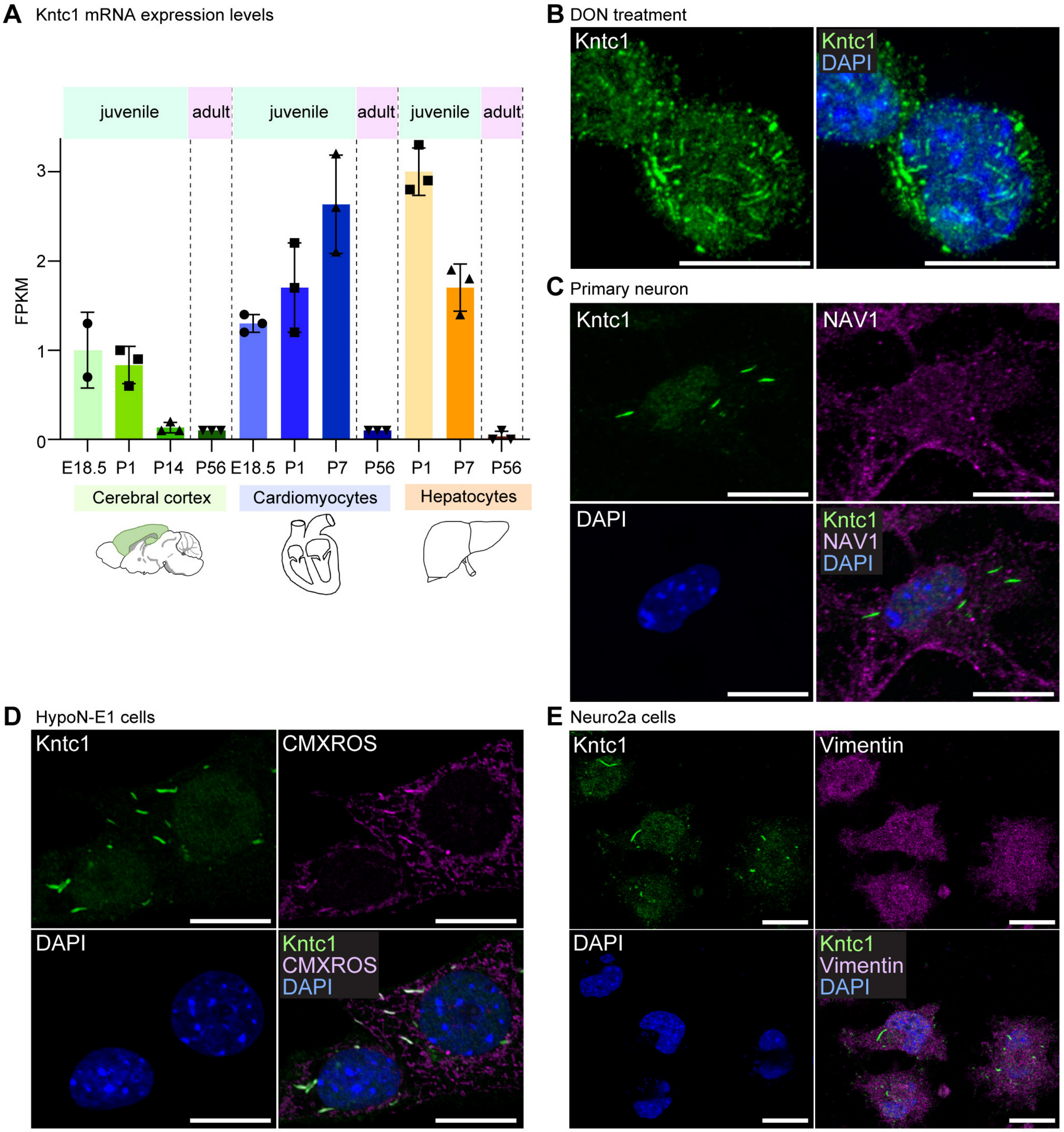
A Kntc1 mRNA expression levels
juvenile
adult
juvenile
adult
juvenile
adult
FPKM
3
2
1
0
E18.5
P1
P14
P56
E18.5
P1
P7
P56
P1
P7
P56
Cerebral cortex
Cardiomyocytes
Hepatocytes
B DON treatment
Kntc1
Kntc1
DAPI
C Primary neuron
Kntc1
NAV1
DAPI
Kntc1
NAV1
DAPI
D HypoN-E1 cells
Kntc1
CMXROS
DAPI
Kntc1
CMXROS
DAPI
E Neuro2a cells
Kntc1
Vimentin
DAPI
Kntc1
Vimentin
DAPI

Fig. S1. Expression and localisation of Kntc1

(A) Expression patterns of Kntc1 in mouse tissues. The expression of Kntc1 is restricted to juvenile stages such as postnatal day 1 (P1) and P7 and is suppressed in adults (P56). FPKM, fragments per kilobase of transcript per million mapped reads.

(B) Immunostaining for Kntc1 in Neuro2a cells treated with DON, a competitive inhibitor of glutamine metabolism. Nuclei were stained with DAPI. Scale bar = 10 µm.

(C) Immunostaining for Kntc1 in mouse primary neurons. Nav1, a microtubule-associated protein, was stained to visualise cell morphology. Scale bar = 10 µm.

(D) Immunostaining for Kntc1 in mouse HypoN-E1 cells, immortalised hypothalamus-originating cells. Scale bar = 10 µm.

(E) Immunostaining for Kntc1 in mouse Neuro2a cells established from neuroblastoma. Scale bar = 10 µm.

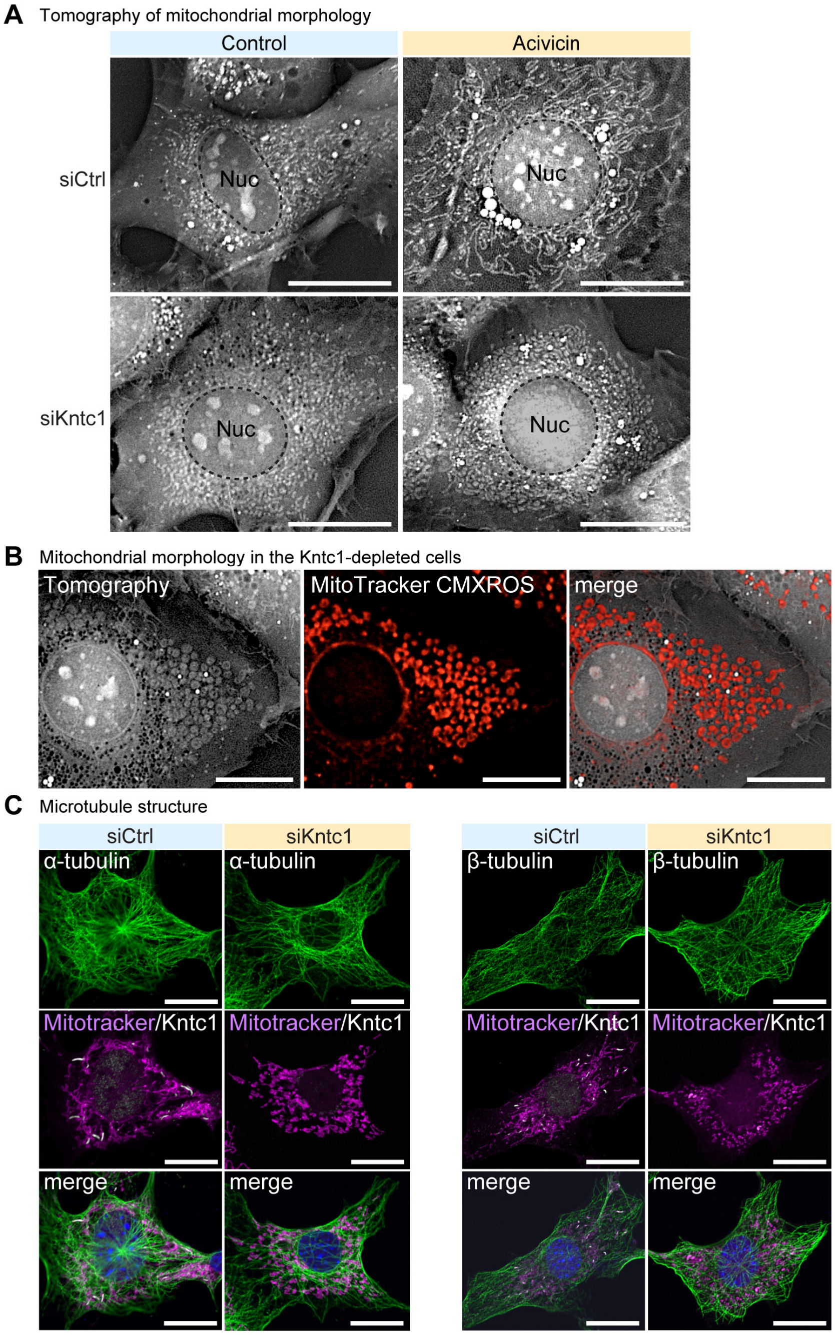

Fig. S2. Kntc1 is responsible for LMS formation

(A) Tomography of NIH3T3 cells to analyze the morphology of mitochondria in Kntc1-depleted cells. Nuc, nucleus. Scale bar = 10 µm.

(B) Simultaneous mitochondrial staining with MitoTracker CMXROS. Scale bar = 10 µm.

(C) Immunostaining of microtubules was performed with a-tubulin and -tubulin antibodies. Kntc1 forming LMS was stained with the Kntc1 antibody. Scale bar = 10 µm.

Disease Models & Mechanisms • Supplementary information

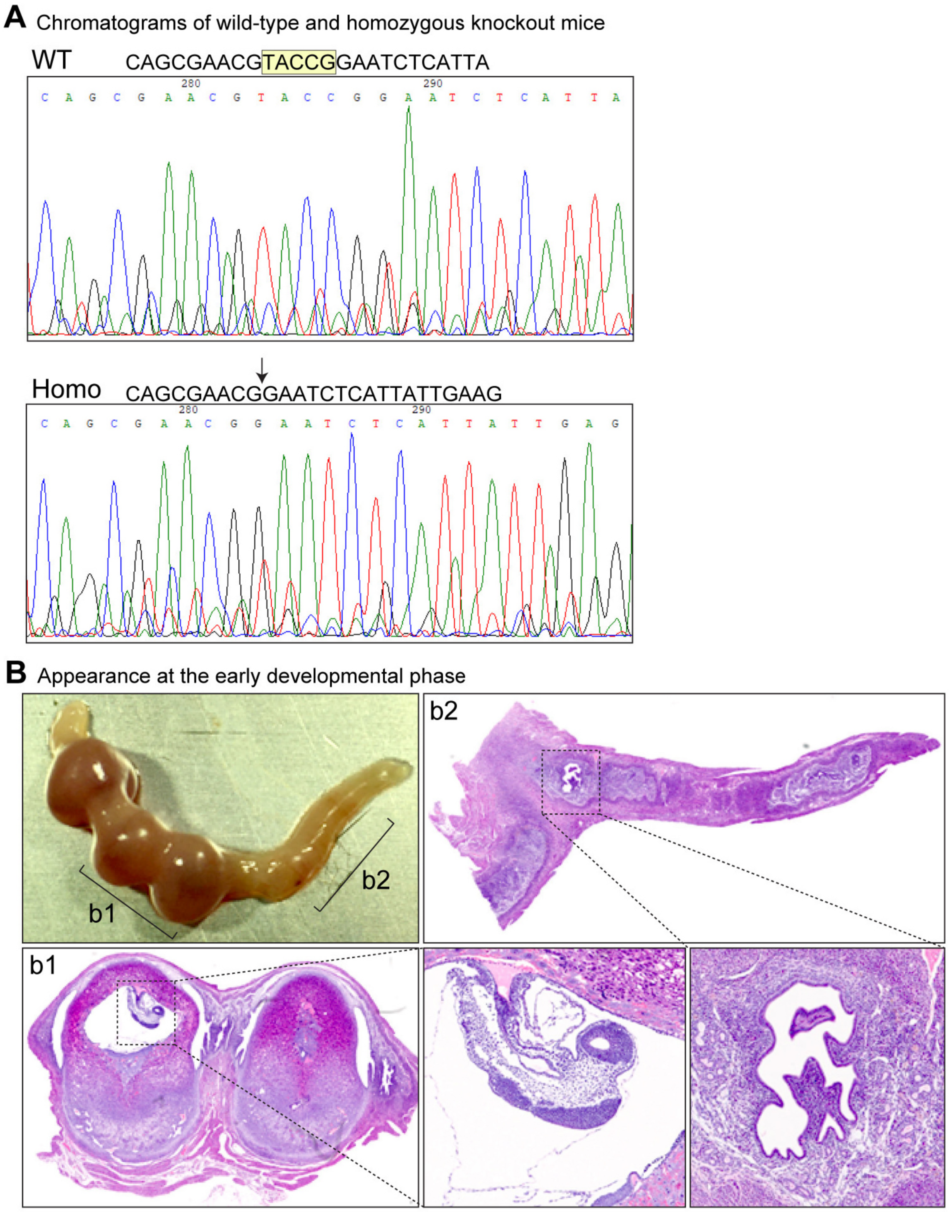

Fig. S3. Kntc1 is indispensable for foetal development.

(A) Chromatograms obtained from wild type and littermate homozygous mutant embryos. The five nucleotides (TACCG) highlighted in yellow were deleted in the mutant allele. Homozygously mutated embryos show the chromatogram pattern from which the five nucleotides were deleted at the position indicated by arrow without overlapping wild type signals.

(B) Appearance of uterus from which foetal tissues were analysed as to genotypes and histology.

(b1,b2) Histological analysis of Kntc1 mutant embryos. The inset b1 shows normally developing embryo with the wild type of genotype. The inset b2 shows typical absorption embryo due to failed embryogenesis at the early developmental phase.

Disease Models & Mechanisms • Supplementary information
